# Comparison of short-and long-term prognostic differences and validation of clinical prediction models between two types of mechanical thrombectomy in patients with acute ischemic stroke aged 75 years and above

**DOI:** 10.3389/fmed.2026.1703596

**Published:** 2026-04-02

**Authors:** Guangying Li, Baorui Zhang, Mingxing Wang, Yonglei Zhu, Sisi Wang, Aixin Liu, Tong Lai

**Affiliations:** 1 Department of Rehabilitation, Lu'an Hospital of Anhui Medical University, Lu'An, China; 2 Department of Rehabilitation, Binzhou People's Hospital Affiliated to Shandong First Medical University, Binzhou, China; 3 Shanghai TCM-integrated Hospital, Shanghai University of TCM, Shanghai, China; 4 Shanghai University of Traditional Chinese Medicine, Shanghai, China; 5 Department of Rehabilitation, Anhui University of Traditional Chinese Medicine Affiliated Lu'an Traditional Chinese Medicine Hospital, Lu'an, China; 6 Department of Surgery, Huicheng Hospital of Traditional Chinese Medicine, Huizhou, China

**Keywords:** Acute ischemic stroke, elderly patients, Inflammatory markers, Mechanical thrombectomy, Prognostic indicator

## Abstract

**Background:**

As the global population ages, the incidence of acute ischemic stroke (AIS) among individuals aged 75 years and older is steadily increasing. Mechanical thrombectomy (MT) has become a standard treatment for AIS, with two commonly employed approaches: bridging thrombectomy (BT) and direct mechanical thrombectomy (DMT). However, the efficacy and safety of these strategies in elderly patients remain inconclusive.

**Methods:**

This single-center retrospective study included AIS patients aged 75 years and older who underwent BT or DMT at Liuan City Hospital of Traditional Chinese Medicine, affiliated with Anhui University of Traditional Chinese Medicine, between December 2020 and August 2024. After propensity score matching (PSM), patients were compared in terms of neurological outcomes (NIHSS, mRS), inflammatory markers (CRP, NLR, SII, SIRI), and complication rates at 24 h, 7 days, and 90 days post-treatment. A predictive model for 90-day functional outcome was developed using univariate and multivariate regression analyses and evaluated for performance.

**Results:**

A total of 87 patients were enrolled, and CRP levels on day 7 after treatment were significantly higher in the BT group than in the DMT group (*P* = 0.008). Multivariate analysis identified time to puncture, NIHSS at 24 h, and CRP at 7 days as independent predictors of 90-day poor functional outcome. Patients with a pre-puncture time >3.25 h had a 6.36-fold higher risk of poor prognosis than those with ≤ 3.25 h. For every 1 mg/L increase in C-reactive protein at 7 days postoperatively, the risk of poor prognosis increased by 2%, with a significant risk rise when the level exceeded 28.175 mg/L. Patients with a NIHSS score >14.5 at 24 h postoperatively had a 2.22-fold higher risk of poor prognosis than those with ≤ 14.5. The predictive model demonstrated high accuracy, with an area under the curve (AUC) of 0.93. And the risk value of 0.319 is the judgment point to distinguish high and low risk groups.

**Conclusion:**

In AIS patients aged 75 years and older, both BT and DMT yielded comparable short- and long-term neurological outcomes. Onset Puncture Time, Postoperative NIHSS at 24 h and CRP at 7 days were key prognostic factors. The developed model may serve as a reliable tool for individualized prognosis assessment and optimization of treatment strategies in elderly stroke patients.

## Introduction

Acute ischemic stroke (AIS) is caused by a disruption in cerebral blood flow, leading to ischemic and hypoxic necrosis and subsequent neurological dysfunction (1). It is the most common and disabling form of acute cerebrovascular disease, posing serious threats to both patient survival and quality of life (2, 3). With global aging on the rise, the proportion of elderly individuals suffering from AIS continues to increase (4). This demographic is characterized by multiple comorbidities and limited physiological reserves, presenting additional challenges to treatment (4).

Mechanical thrombectomy (MT) has become a standard treatment for AIS, as it enables rapid revascularization in cases of large vessel occlusion and significantly improves neurological outcomes (5, 6). However, the effectiveness and safety of MT in elderly patients remain subjects of ongoing debate (7). Clinically, two primary approaches are employed: direct mechanical thrombectomy (DMT) and bridging thrombectomy (BT). BT was defined as intravenous thrombolysis followed by mechanical thrombectomy: after the diagnosis of large vessel occlusion (LVO) by cranial MRI/head and neck CTA, rtPA (0.9 mg/kg, maximum 90 mg) was intravenously administered within 4.5 h of symptom onset, and mechanical thrombectomy was performed within 2 h after the end of thrombolysis if the neurological function did not improve significantly (NIHSS score reduction < 10% or aggravation). DMT was defined as mechanical thrombectomy without preoperative intravenous thrombolysis, which was performed immediately after the diagnosis of LVO. The decision of thrombectomy/lysis was made by the multidisciplinary team (neurologist + interventional neurologist) based on the patient's symptom onset time, imaging results and neurological function status. The definition of time points was unified: symptom-to-onset time was the interval from the onset of neurological deficit symptoms to hospital admission; symptom-to-puncture time (OPT) was the interval from symptom onset to femoral artery puncture for thrombectomy. While recent studies suggest that both approaches are effective in AIS patients (8–10), no consensus exists regarding the optimal strategy for the elderly (11, 12).

Compared with DMT, BT allows for earlier initiation of thrombolysis, and some patients may achieve partial or complete recanalization through intravenous thrombolysis alone, potentially reducing the time and risk associated with mechanical intervention (13–15). BT also provides more flexible treatment options for patients with varying degrees of vessel occlusion, particularly for those without a confirmed diagnosis of large vessel occlusion but who are eligible for intravenous thrombolysis (16). However, elderly patients face higher risks of hemorrhage and lower recanalization rates with intravenous thrombolysis, which may make DMT a more suitable alternative (17, 18). Despite this, the comparative benefits of BT vs. DMT in elderly patients, particularly regarding short- and long-term outcomes, remain poorly understood.

This study focused on AIS patients aged 75 years and older, with 24-h and 7-day time points used to assess short-term outcomes and a 90-day time point used to assess long-term outcomes. The aim was to compare the effectiveness of DMT and BT in this population and to develop and validate a clinical prediction model for 90-day functional outcomes based on significant independent variables. By clarifying the relative merits of these two approaches and offering a reliable prognostic model, this study seeks to optimize treatment strategies and improve outcomes in elderly AIS patients.

## Methods

### Participants

This retrospective study covers AIS patients aged 75 and older who received DMT or BT at the Lu'an City Hospital of Traditional Chinese Medicine, affiliated with Anhui University of Traditional Chinese Medicine, from December 1, 2020, to August 31, 2024. Ethical approval for this study was obtained from the Ethics Committee of Lu‘an Hospital of Traditional Chinese Medicine. (No: LASZYY-LL-2024030) and was conducted in accordance with the Declaration of Helsinki. Each patient signed an informed consent form. All patient selections were conducted by an experienced neurologist and an assistant.

Inclusion criteria: (1) NIHSS score ≥6 at admission; (2) Diagnostic confirmation by cranial MRI and head/neck CTA; (3) Age ≥75 years; (4) OPT ≤ 6 h; (5) Availability of NIHSS and inflammatory marker data at 24 h and 7 days postoperatively; (6) Postoperative standard care including blood pressure control, thrombosis prevention, and adjunctive treatment (CV8 acupoint patching); (7) Complete 90-day follow-up data.

Exclusion criteria: (1) Presence of confounding conditions such as malignancy, severe hepatic, renal, or cardiac dysfunction; (2) Interruption of follow-up due to unrelated medical issues; (3) Stroke history or significant sequelae within the previous 6 months.

### Data Collection

Baseline data collected included gender, age, time of onset, blood glucose on admission, NIHSS score, history of hypertension, diabetes, atrial fibrillation, prior cerebral infarction, and oral anticoagulant use. Surgical information covered the affected vessel, treatment method, and recanalization success. The short-term outcome was based on NIHSS and laboratory indicators and calculated inflammation index, including Neutrophil count, lymphocyte count, Platelet count, Monocyte count, (XN-9000 Fully automatic blood analyzer, Sysmex) and CRP (H7600 Biochemical analyzer, transmittance turbidimetry), the time of sampling was 24 h after surgery and morning on day 7 after surgery, and the patient was in a fasting state. Long-term outcomes were assessed by 90-day mRS score, with patients classified into favorable (mRS < 3) or unfavorable (mRS ≥3) outcome groups. Complications recorded included reinfarction, symptomatic intracerebral hemorrhage, and stroke-associated pneumonia. Door-to-groin time (DGT) was defined as the interval from hospital admission to femoral artery puncture for mechanical thrombectomy. All time indicators were recorded by the special research nurse and verified by the medical record.

### Inflammatory marker definitions

NLR = neutrophil count/lymphocyte count.

SII = platelet count × neutrophil count/lymphocyte count.

SIRI = (neutrophil count/lymphocyte count)/monocyte count (19).

### Study cohort

Patients were categorized into two groups based on whether they received intravenous thrombolysis prior to mechanical thrombectomy: the BT group and the direct thrombectomy DMT group. Baseline and clinical data were collected for both groups. After propensity score matching, comparative analyses were conducted within each group and between the two groups to evaluate treatment effects and outcomes.

### Statistical analysis

The distribution of variables was first assessed for normality. Continuous variables following a normal distribution were compared using independent-samples *t*-tests and presented as mean ± standard deviation (SD). Non-normally distributed variables were compared using the Mann–Whitney U test and presented as median (interquartile range, IQR). Categorical variables were analyzed using the Chi-square test (χ^2^) or Fisher's exact test, as appropriate.

To minimize the impact of inter-group baseline confounding factors, this study employed propensity score matching (PSM) to align patients between the BT and direct DMT groups. The specific matching strategy implemented 1:1 nearest neighbor matching, with a clamp width of 0.2 times the standard deviation of propensity scores (PS). Only patients with matching PS values within this range from either the treatment group (BT) or control group (DMT) were paired, ensuring balanced baseline characteristics between the groups after matching.

For outcome prediction modeling, patients were stratified based on their 90-day (mRS) scores. The dataset was randomly split into a training set (70%) and a validation set (30%). Variable selection was performed using univariate logistic regression, followed by bootstrap resampling (1,000 iterations) to ensure stability. Variables with significance (*P* < 0.1) were included in multivariate logistic regression to construct the final predictive model.

Model performance was assessed using the following metrics:

• Receiver Operating Characteristic (ROC) curve analysis and Area Under the Curve (AUC)

• Nomogram construction for clinical application

• Calibration curve, Hosmer-Lemeshow goodness-of-fit test, Cox-Snell R^2^, and Likelihood Ratio Test

• Confusion matrix to assess sensitivity, specificity, predictive accuracy

All statistical analyses were conducted using SPSS Statistics 27.0 and MedCalc, while visualization was performed using R version 4.4.3. A two-sided *P*-value < 0.05 was considered statistically significant.

## Results

### Baseline characteristics

**Figure 1:**
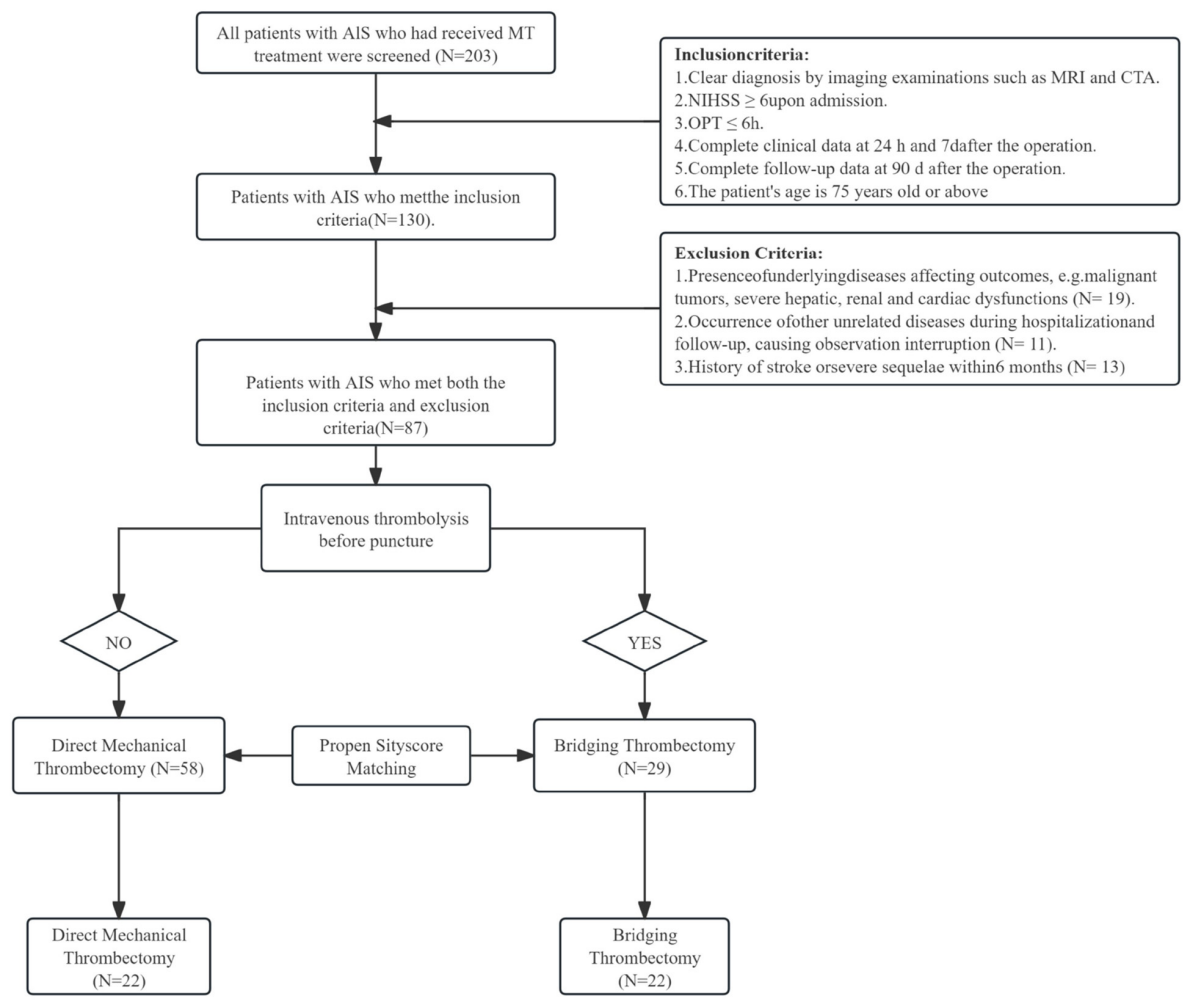
Flow chart for the selection of AIS patients receiving MT and DMT treatment.

A total of 87 patients who met the inclusion criteria were enrolled in this study (Figure 1). Of these, 29 patients received bridging thrombectomy, and 58 patients received direct mechanical thrombectomy. After 1:1 propensity score matching, 22 patients remained in each group for comparative analysis.

Statistical comparisons of baseline variables between the matched groups showed no significant differences, indicating good balance and comparability across demographic and clinical characteristics (Table 1).

**Table 1:** The patient's tendency to match the baseline data before and after scoring.

Variable	Before PSM					After PSM				
	BT (*n* = 29)	DMT (*n* = 58)	Statistic	*P*	SMD	BT (*n* = 22)	DMT (*n* = 22)	Statistic	*P*	SMD
Age (y), mean ± SD	79.72 ± 4.74	80.36 ± 4.76	t = -0.590	0.557	0.134	79.50 ± 4.33	80.36 ± 4.86	t = −0.623	0.537	0.178
OPT (h), mean ± SD	3.60 ± 1.04	3.34 ± 1.02	t = 1.143	0.256	−0.261	3.64 ± 0.95	3.48 ± 0.78	t = 0.606	0.548	−0.204
DGT (h), mean ± SD	0.41 ± 0.16	0.37 ± 0.23	t = 0.741	0.312	0.226	0.38 ± 0.15	0.36 ± 0.13	t = 0.445	0.097	0.314
ABG (mmol/l), mean ± SD	7.33 ± 1.58	7.72 ± 2.42	t =−0.768	0.445	0.157	7.34 ± 1.72	7.14 ± 2.28	t = 0.329	0.744	−0.088
CRP (mg/L), M (Q_1_, Q_3_)	11.40 (2.64, 20.16)	20.85 (3.54, 38.16)	Z =−1.771	0.438	0.124	16.40 (2.64, 20.16)	18.85 (3.54, 30.16)	Z = −0.409	0.699	−0.072
NIHSS, Mean ± SD	16.52 ± 4.66	18.53 ± 6.20	t = -1.546	0.126	0.325	17.41 ± 4.45	17.14 ± 6.24	t = 0.167	0.868	−0.044
mRS ≤ 2, *n* (%)										
Yes	13 (44.83)	27 (46.55)	χ^2^ = 0.209	0.647	0.112	7 (31.82)	8 (36.36)	χ^2^ = 0.101	0.750	0.048
No	16 (55.17)	31 (53.45)				15 (68.18)	14 (63.64)			
Gender, *n* (%)										
Male	17 (58.62)	29 (50.00)	χ^2^ = 0.577	0.448	−0.172	13 (59.09)	11 (50.00)	χ^2^ = 0.367	0.545	−0.182
Female	12 (41.38)	29 (50.00)			0.172	9 (40.91)	11 (50.00)			0.182
Hypertension, *n* (%)										
Yes	3 (10.34)	14 (24.14)	χ^2^ = 2.339	0.126	0.322	3 (13.64)	3 (13.64)	χ^2^ = 0.000	1.000	0.000
No	26 (89.66)	44 (75.86)			−0.322	19 (86.36)	19 (86.36)			0.000
DM, *n* (%)										
Yes	24 (82.76)	46 (79.31)	χ^2^ = 0.146	0.702	−0.085	18 (81.82)	18 (81.82)	χ^2^ = 0.000	1.000	0.000
No	5 (17.24)	12 (20.69)			0.085	4 (18.18)	4 (18.18)			0.000
History of cerebral infarction, *n* (%)										
Yes	23 (79.31)	37 (63.79)	χ^2^ = 2.175	0.140	−0.323	19 (86.36)	14 (63.64)	χ^2^ = 3.030	0.082	−0.472
No	6 (20.69)	21 (36.21)			0.323	3 (13.64)	8 (36.36)			0.472
OAs, *n* (%)										
Yes	26 (89.66)	55 (94.83)	χ^2^ = 0.201	0.654	0.234	22 (100.00)	20 (90.91)	χ^2^ = 0.524	0.469	−0.316
No	3 (10.34)	3 (5.17)			−0.234	0 (0.00)	2 (9.09)			0.316
Affected vessel, *n* (%)										
Right MCA	5 (17.24)	15 (25.86)	χ^2^ = 4.125	0.389	0.197	3 (13.64)	4 (18.18)	–	0.857	0.118
Right ICA	6 (20.69)	12 (20.69)			0.000	5 (22.73)	2 (9.09)			−0.474
Left MCA	10 (34.48)	15 (25.86)			−0.197	8 (36.36)	10 (45.45)			0.183
Left ICA	6 (20.69)	6 (10.34)			−0.340	4 (18.18)	4 (18.18)			0.000
BA	2 (6.90)	10 (17.24)			0.274	2 (9.09)	2 (9.09)			0.000
AF, *n* (%)										
Yes	12 (41.38)	20 (34.48)	χ^2^ = 0.395	0.529	−0.145	10 (45.45)	10 (45.45)	χ^2^ = 0.000	1.000	0.000
No	17 (58.62)	38 (65.52)			0.145	12 (54.55)	12 (54.55)			0.000
Successful recanalization, *n* (%)										
0	22 (75.86)	49 (84.48)	χ^2^ = 0.957	0.328	0.238	18 (81.82)	19 (86.36)	χ^2^ = 0.000	1.000	0.132
1	7 (24.14)	9 (15.52)			−0.238	4 (18.18)	3 (13.64)			−0.132

OPT, Onset-to-puncture time; ABG, Admission blood glucose; NIHSS, National Institutes of Health Stroke Scale; OAs, Oral anticoagulants; DM, Diabetes mellitus; AF, Atrial fibrillation; MCA, Middle Cerebral Artery; ICA, Internal Carotid Artery; BA, Basilar Artery; SIRI, Systemic Inflammation Response Index; SII, Systemic Inflammatory Index; NLR, Neutrophil-to-lymphocyte ratio; CRP, C-reactive protein; mRS, Modified Rankin Scale; M, Median; SD, Standard deviation.

### Within-group comparisons

**Figure 2:**
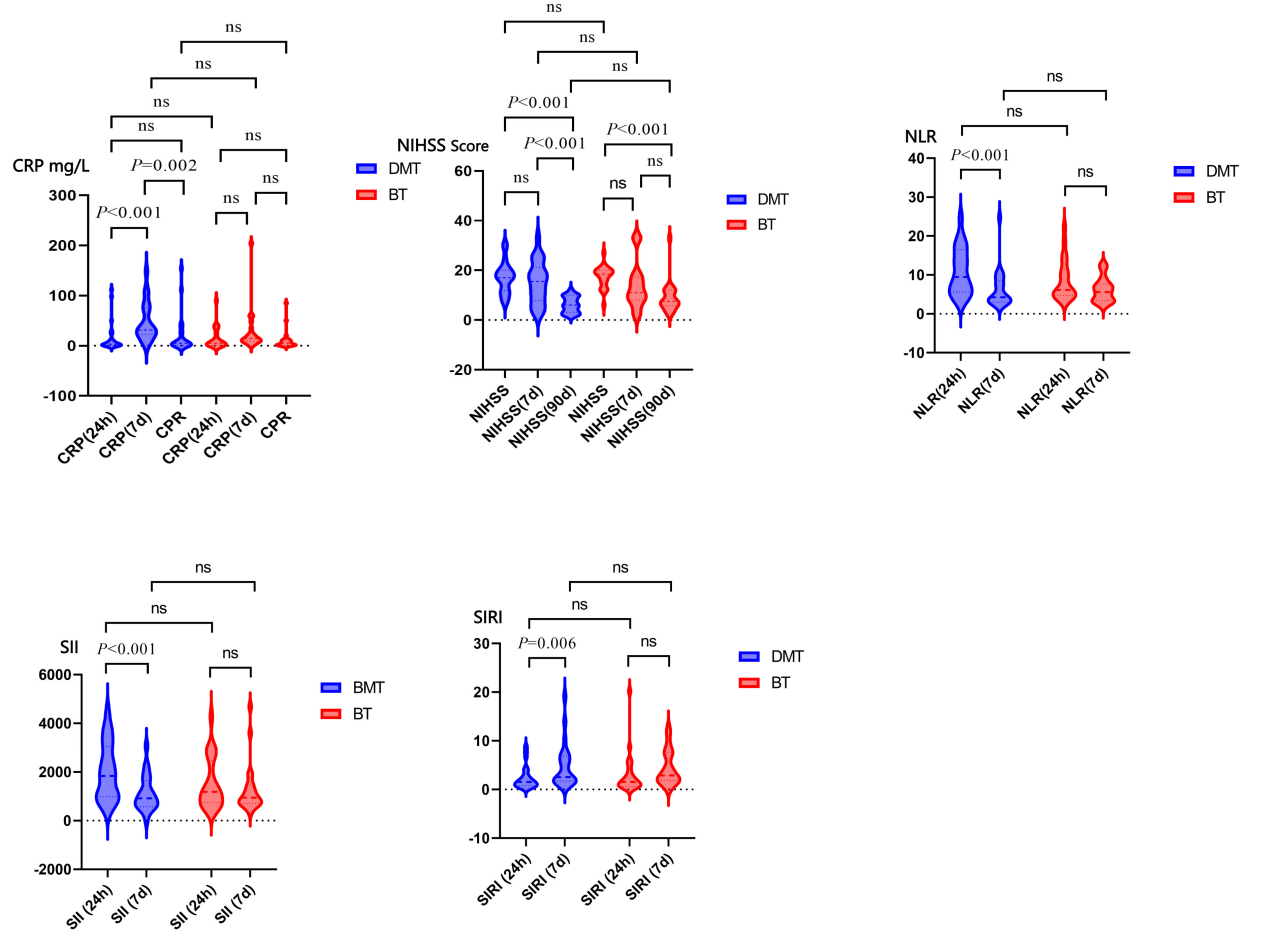
The difference between the two groups of patients at 24h and 7d after surgery. Abbreviations: NIHSS, national institutes of health stroke scale; SIRI, systemic inflammation response index; SII, systemic inflammatory index; NLR, neutrophil-to-lymphocyte ratio; CRP, C-reactive protein.

In the BT group, a statistically significant increase in CRP was observed from 24 h to 7 days postoperatively (*P* = 0.003), while NIHSS scores showed significant improvement over the same period (*P* < 0.001). Changes in NLR, SII, and SIRI were not statistically significant. In the DMT group, NIHSS scores significantly decreased from 24 h to 7 days post-treatment (*P* < 0.001). Inflammatory indicators demonstrated the following trends: NLR and SII significantly decreased (*P* < 0.001), while SIRI increased significantly (*P* = 0.006). CRP levels showed no statistically significant change (Figure 2).

### Between-group comparisons

There were no statistically significant differences between the two groups regarding short-term neurological outcomes (NIHSS scores) or inflammatory markers (NLR, SII, SIRI) at both 24 h and 7 days postoperatively. However, at 7 days, CRP levels were significantly lower in the DMT group compared to the BT group (*P* = 0.008), indicating a potentially greater systemic inflammatory response in the BT group. Long-term outcomes at 90 days, including mRS scores, rates of symptomatic intracranial hemorrhage, stroke-related pneumonia, and reinfarction, showed no statistically significant differences between the groups (Table 2, Figure 2).

**Table 2:** Outcome data of AIS patients with DMT and BT.

Variables	BT (*n* = 22)	DMT (*n* = 22)	Statistic	*P*
24 h after surgery				
NIHSS, M (Q_1_, Q_3_)	18.50 (14.25, 20.00)	19.00 (13.25, 21.50)	Z = −0.22	0.813
CRP (mg/L), M (Q_1_, Q_3_)	5.27 (0.89, 17.67)	3.09 (0.62, 11.38)	Z = −0.43	0.656
NLR1, M (Q_1_, Q_3_)	9.48 (5.99, 15.81)	6.13 (4.76, 10.63)	Z = −1.42	0.152
SII, M (Q_1_, Q_3_)	1,835.11 (1,018.15, 2,910.16)	1,182.72 (790.31, 2,215.52)	Z = −1.56	0.116
SIRI, M (Q_1_, Q_3_)	1.54 (0.84, 2.88)	1.56 (0.69, 3.36)	Z = −0.14	0.879
7 days after surgery				
NIHSS, M (Q_1_, Q_3_)	11.00 (8.00, 16.75)	15.50 (8.50, 19.75)	Z = −0.86	0.384
mRS ≤ 2, *n* (%)				
Yes	8 (36.4)	9 (40.9)	χ^2^ = 0.096	0.757
No	14 (63.6)	13 (59.1)		
CRP (mg/L), M (Q_1_, Q_3_)	31.35 (24.82, 72.93)	14.46 (9.54, 24.14)	Z = −2.64	0.008
NLR2, M (Q_1_, Q_3_)	4.29 (3.10, 8.05)	5.61 (3.45, 7.37)	Z = −0.47	0.630
SII, M (Q_1_, Q_3_)	914.94 (590.55, 1,535.73)	945.37 (709.92, 1,411.73)	Z = −0.43	0.656
SIRI, M (Q_1_, Q_3_)	2.55 (1.79, 6.70)	2.88 (1.86, 7.42)	Z = -0.08	0.925
90 Days after surgery				
NIHSS, M (Q_1_, Q_3_)	6.23 (4.80, 7.66)	9.00 (6.19,11.81)	Z = -1.72	0.086
mRS ≤ 2, *n* (%)				
Yes	12 (54.55)	10 (45.45)	χ^2^ = 0.36	0.546
No	10 (45.45)	12 (54.55)		
Reinfarction, *n* (%)				
Yes	1 (4.55)	1 (4.55)	χ^2^ = 0.00	1.000
No	21 (95.45)	21 (95.45)		
sICH, *n* (%)				
Yes	3 (13.64)	1 (4.55)	χ^2^ = 0.28	0.600
No	19 (86.36)	21 (95.45)		
Stroke-associated pneumonia, *n* (%)				
Yes	15 (68.18)	13 (59.09)	χ^2^ = 0.39	0.531
No	7 (31.82)	9 (40.91)		

NIHSS, National Institutes of Health Stroke Scale; SIRI, Systemic Inflammation Response Index; SII, Systemic Inflammatory Index; NLR, Neutrophil-to-lymphocyte ratio; CRP, C-reactive protein; mRS, Modified Rankin Scale; sICH, symptomatic Intracerebral Hemorrhage; M, Median; Q1, 1st Quartile; Q3, 3rd Quartile.

### Predictive model for 90-day outcomes

To identify independent predictors of 90-day functional outcomes, univariate logistic regressionanalysis was performed. Variables with *P* < 0.1 were selected for further analysis. Using 1,000 bootstrap iterations, the most robust predictors were identified:

• Onset-to-puncture time.

• NIHSS score at 24 h post-treatment.

• CRP level at 7 days post-treatment.

These variables were then included in a multivariate logistic regression model, and all three were found to be independently associated with poor 90-day outcomes (Table 3).

**Table 3:** Univariate and Multivariate Logistic Regression Analysis for 90-Day Poor Outcome.

Variables	Univariate OR (95% CI)	*P*	Multivariate OR (95% CI)	*P*
Age (y)	1.04 (0.93–1.16)	0.503	–	–
OPT (h)	1.98 (1.14–3.44)	0.016	6.36 (1.32–30.61)	0.021
ABG (mmol/l)	1.36 (1.01–1.82)	0.042	1.13 (0.80–1.58)	0.486
Admission NIHSS score	1.12 (1.02–1.24)	0.024	0.71 (0.50–1.03)	0.068
NIHSS at 24h	1.35 (1.14–1.58)	< .001	2.22 (1.23–3.99)	0.008
NIHSS at Day 7	7,398.00 (0.00–Inf)	0.997	–	–
CRP at 24h	0.98 (0.96–1.01)	0.169	–	–
CRP at Day 7	1.01 (1.00–1.03)	0.094	1.02 (1.00–1.04)	0.040
NLR at 24h	1.06 (0.97–1.15)	0.197	–	–
NLR at Day 7	1.06 (0.97–1.15)	0.177	–	–
SII at 24h	1.00 (1.00–1.00)	0.153	–	–
SII at Day 7	1.00 (1.00–1.00)	0.273	–	–
SIRI at 24h	1.03 (0.92–1.16)	0.607	–	–
SIRI at Day 7	1.06 (0.98–1.15)	0.146	–	–
Gender, *n* (%)			–	–
0	1.00 (Reference)		–	–
1	2.85 (0.91–8.93)	0.172	–	–
Gender, n (%)			–	–
Male	1.00 (Reference)		–	–
Female	0.95 (0.34–2.66)	0.916	–	–
Hypertension, *n* (%)				
Yes	1.00 (Reference)		–	–
No	2.33 (0.54–10.06)	0.256	–	–
DM, *n* (%)				
Yes	1.00 (Reference)		–	–
No	1.18 (0.33–4.19)	0.796	–	–
History of Cerebral Infarction, *n* (%)				
Yes	1.00 (Reference)		–	–
No	1.22 (0.42–3.58)	0.715	–	–
OAs, *n* (%)				
Yes	1.00 (Reference)		–	–
No	0.74 (0.12–4.80)	0.756	–	–
Affected vessel, *n* (%)				
Right MCA	1.00 (Reference)		–	–
Right ICA	3.60 (0.71–18.25)	0.122	–	–
Left MCA	1.57 (0.34–7.22)	0.559	–	–
Left ICA	0.75 (0.10–5.47)	0.777	–	–
BA	7.87 (1.10–56.12)	0.159	–	–
AF, *n* (%)				
Yes	1.00 (Reference)		–	–
No	0.68 (0.25–1.90)	0.467	–	–

OPT, Onset-to-puncture time; ABG, Admission blood glucose; NIHSS, National Institutes of Health Stroke Scale; OAs, Oral anticoagulants; DM, Diabetes mellitus; AF, Atrial fibrillation; MCA, Middle Cerebral Artery; ICA, Internal Carotid Artery; BA, Basilar Artery; SIRI, Systemic Inflammation Response Index; SII, Systemic Inflammatory Index; NLR, Neutrophil-to-lymphocyte ratio; CRP, C-reactive protein; OR, odds ratio.

### Model performance

**Figure 3:**
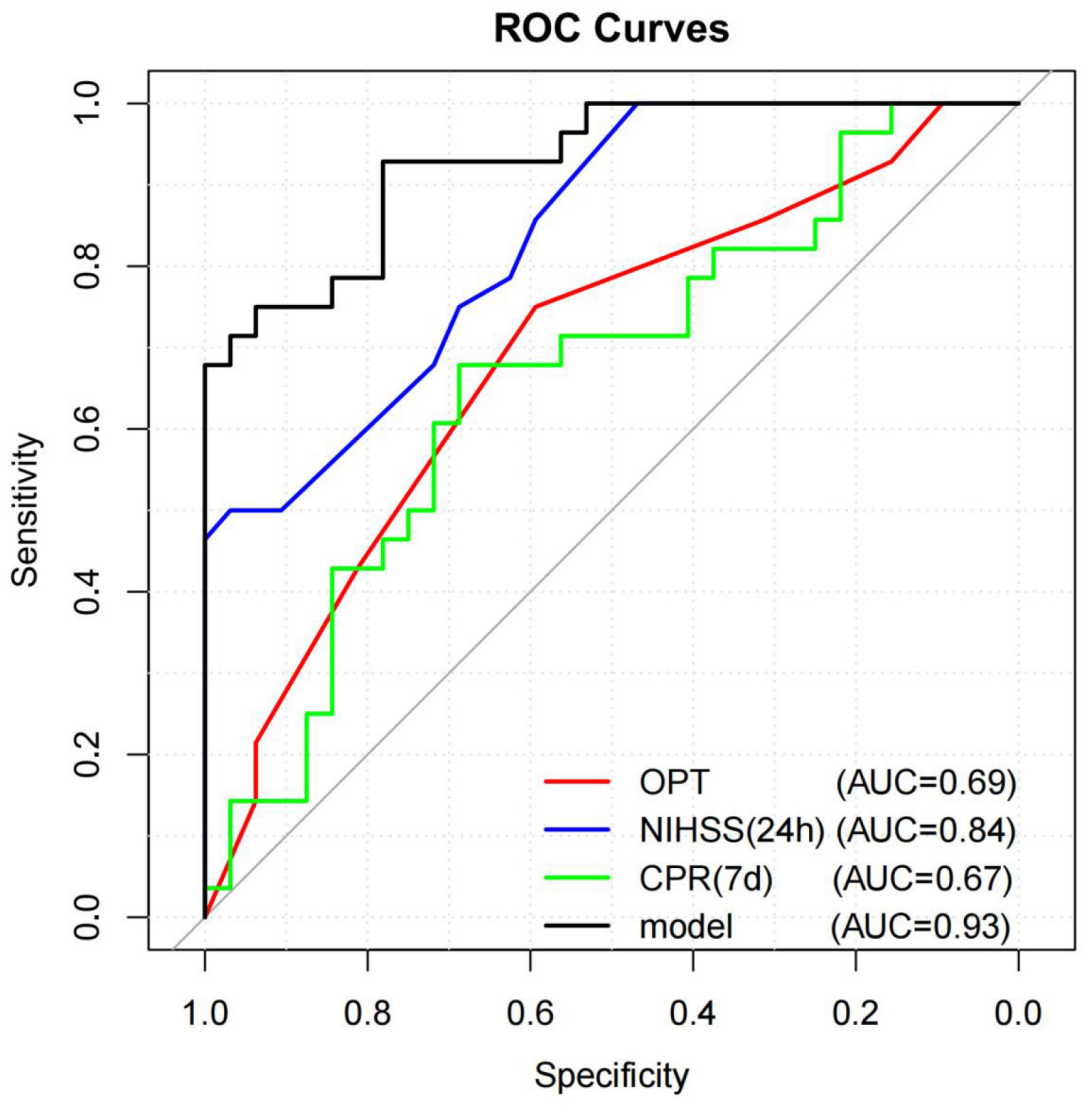
ROC curve and AUC value of risk factors for 90-day poor outcome. An AUC value closer to 1 indicates better predictive accuracy, while an AUC of 0.5 represents no discriminative value. Abbreviations: OPT, onset-to-puncture time; NIHSS, national institutes of health stroke scale; CRP, C-reactive protein; AUC, area under the curve.

ROC curve analysis was used to evaluate the predictive accuracy of each independent variable and the overall model: AUC for onset-to-puncture time: 0.69 (95% CI: 0.46–0.79); AUC for NIHSS at 24h: 0.84 (95% CI: 0.74–0.97); AUC for CRP at day 7: 0.67 (95% CI: 0.56–0.83); AUC for composite model: 0.93 (95% CI: 0.86–0.99); The significantly higher AUC of the composite model compared to individual predictors indicates improved predictive power when combining multiple variables. This study identified three core prognostic predictors through the optimal Jordan index and quantified their association with poor prognosis (mRS 90d≥3) using multivariate logistic regression. The nomogram model established risk thresholds as follows: The optimal cutoff for pre-puncture time was 3.25 h, with multivariate analysis showing it to be an independent risk factor (OR = 6.36; 95% CI: 1.32–30.61), meaning patients with pre-puncture time> 3.25 h had 6.36 times higher risk of poor prognosis compared to those ≤ 3.25 h. For postoperative 7-day CRP levels, the best cutoff was 28.175 mg/L, demonstrating significant association (OR = 1.02; 95% CI: 1.00–1.04). This indicates a 2% increase in poor prognosis risk per unit elevation of CRP after 7 days, with a marked rise when levels exceed 28.175 mg/L. Regarding postoperative 24-h NIHSS scores, the optimal cutoff was 14.5 points, confirmed by multivariate regression as an independent predictor (OR = 2.22; 95% CI: 1.23–3.99). Patients scoring> 14.5 points showed 2.22 times higher risk of poor prognosis than those ≤ 14.5 points. The predictive model constructed based on the three aforementioned indicators demonstrates an optimal risk cutoff value of 0.319 (risk scale: 0–1). In clinical practice, this threshold enables patient stratification into two groups: “high-risk” (risk> 0.319) and “low-risk” (risk ≤ 0.319), with the high-risk group receiving enhanced monitoring protocols or preemptive interventions to improve prognosis. The results confirm the model's high predictive accuracy, as evidenced by its excellent AUC value on the ROC curve, which effectively distinguishes between high-risk and low-risk patients. Furthermore, the predictive performance evaluated through ROC analysis significantly enhances the clinical applicability of this model (Figure 3, Table 4).

**Table 4:** Confusion matrix of risk factors and models.

Variables	AUC (95%CI)	Accuracy (95%CI)	Sensitivity (95%CI)	Specificity (95%CI)	PPV (95%CI)	NPV (95%CI)	Cut off
NIHSS (24h)	0.84 (0.74–0.93)	0.72 (0.59–0.83)	0.47 (0.30–0.64)	0.97 (0.88–1.00)	0.88 (0.79–0.94)	0.62 (0.48–0.76)	14.5
CRP (7d)	0.67 (0.53–0.81)	0.68 (0.55–0.80)	0.69 (0.53–0.85)	0.68 (0.51–0.85)	0.71 (0.55–0.87)	0.66 (0.48–0.83)	28.175
OPT	0.69 (0.56–0.83)	0.67 (0.53–0.78)	0.59 (0.42–0.76)	0.75 (0.59–0.91)	0.73 (0.56–0.90)	0.62 (0.45–0.78)	3.25
Model	0.93 (0.86–0.99)	0.85 (0.73–0.93)	0.78 (0.64–0.92)	0.93 (0.83–1.00)	0.93 (0.83–1.00)	0.79 (0.65–0.93)	0.319

OPT, Onset-to-puncture time; NIHSS, National Institutes of Health Stroke Scale; CRP, C-reactive protein; AUC, Area Under The Curve; PPV, Positive Predictive Value; NPV, Negative Predictive Value; OR, Odds Ratio.

### Nomogram construction and evaluation

**Figure 4:**
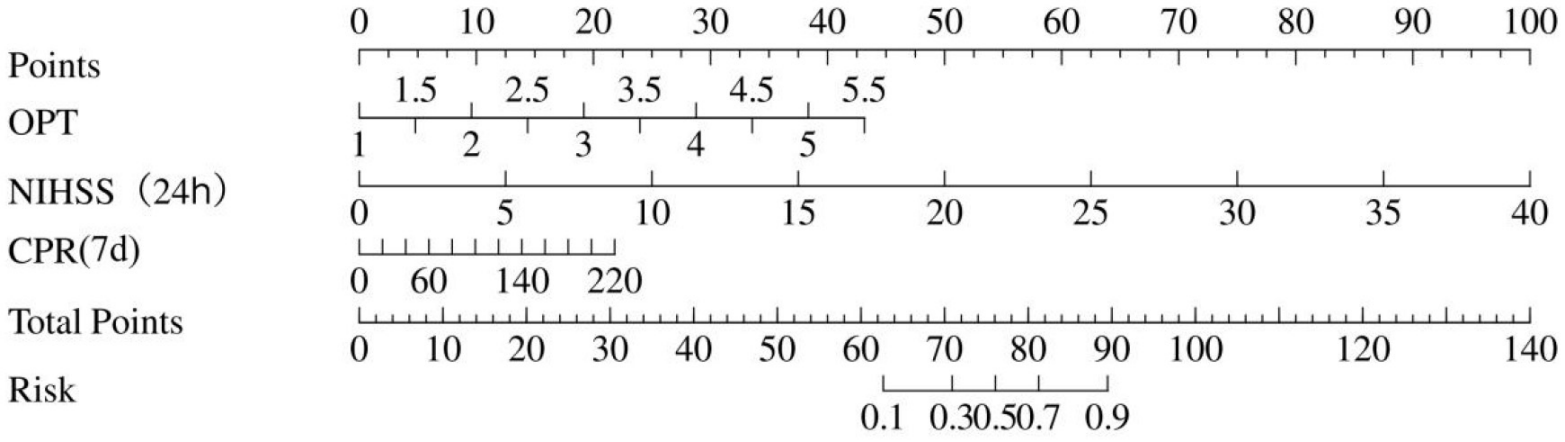
Nomogram for predicting a 90-day poor outcome. The total points of all variables could be converted into the predicted probability of 90-day poor outcome, providing a quantitative and clinically convenient tool for individual prognosis assessment. Abbreviations: OPT, onset-to-puncture time; NIHSS, national institutes of health stroke scale; CRP, C-reactive protein.

A nomogram was developed based on the multivariate regression model to provide a user-friendly clinical tool for individualized 90-day outcome prediction. Each predictor was assigned a weighted score based on its regression coefficient. The total score corresponds to a predicted probability of a poor functional outcome (Figure 4).

**Figure 5:**
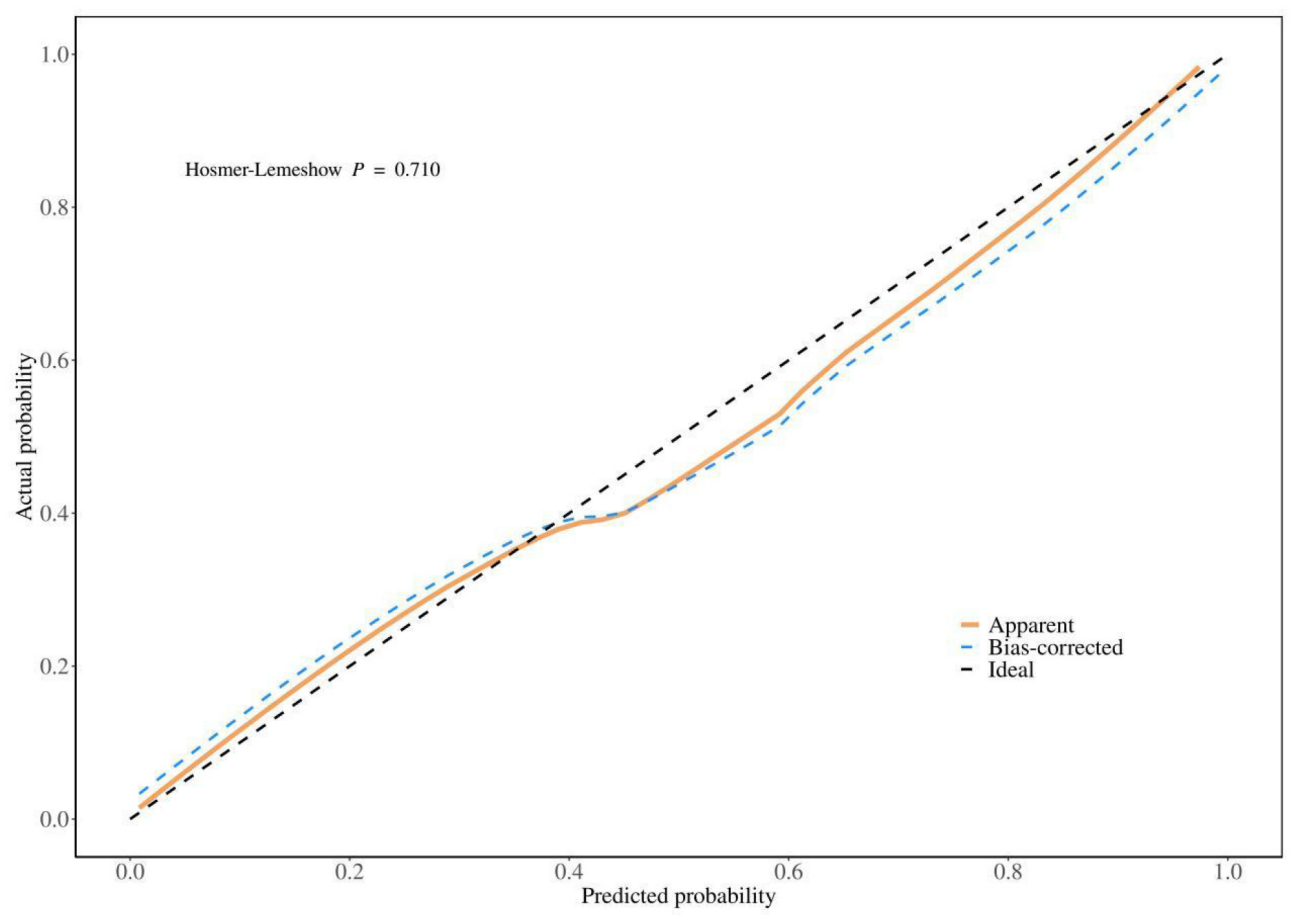
Calibration curve of the 90-day poor outcome model. The calibration curve was used to evaluate the consistency between the predicted probability and the actual observed probability of 90-day poor functional outcome. The diagonal line represents an ideal perfect prediction.

Model calibration showed strong agreement between predicted and observed outcomes, with a C-index of 0.875, indicating high predictive accuracy. The Hosmer–Lemeshow test showed good model fit (*P* = 0.710), while the Cox–Snell likelihood ratio test was statistically significant (χ^2^ = 28.534, *P* < 0.001), confirming the model's goodness of fit. The Cox–Snell R^2^ value was 0.749, suggesting the model explains approximately 74.9% of the variation in outcomes (Figure 5).

**Figure 6:**
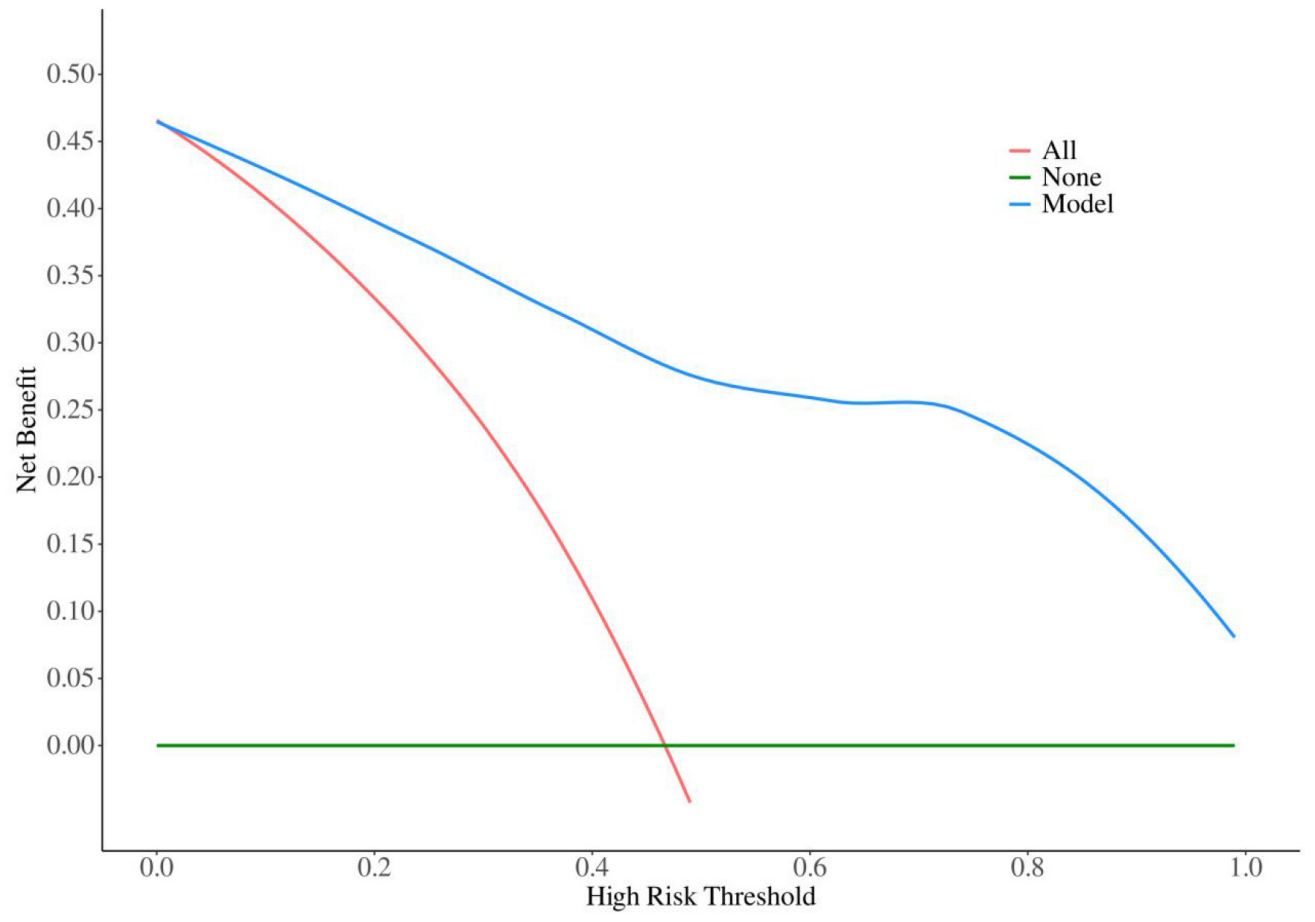
DCA curve of 90-day poor outcome prediction model. DCA was performed to evaluate the clinical net benefit of the prediction model for 90-day poor outcome across different threshold probabilities.

Decision Curve Analysis (DCA) further confirmed the clinical net benefit of the model, demonstrating its potential to guide personalized treatment decisions in elderly AIS patients (Figure 6).

## Discussion

This study aimed to compare the short- and long-term outcomes of DMT vs. BT in patients aged 75 years and older with AIS. By assessing clinical outcomes at 24 h, 7 days, and 90 days postoperatively, this study provides insight into the effectiveness and prognostic impact of these two endovascular strategies in the elderly population.

First, the results revealed no significant differences in short-term neurological recovery between the two groups, as measured by the NIHSS, which aligns with previous findings (20, 21). However, at 7 days postoperatively, CRP levels were significantly higher in the BT group compared to the DMT group (*P* = 0.008), suggesting a potentially stronger inflammatory response associated with bridging therapy. Prior studies have identified CRP as a prognostic biomarker following mechanical thrombectomy, with elevated levels being significantly correlated with poor 90-day outcomes (22). Notably, there is a paucity of comparative research specifically focusing on elderly patients undergoing BT vs. DMT, and this study helps address that gap by providing valuable data. Other inflammatory markers such as NLR, SII, and SIRI showed no significant intergroup differences, indicating potential individual variability or time-dependent effects in immune responses to different treatment strategies (23).

The baseline comorbidities (hypertension, diabetes, atrial fibrillation) of the two groups were not significantly different (*P* > 0.05), and the univariate and multivariate logistic regression analysis also showed that comorbidities were not independent predictors of 90-day poor prognosis (*P* > 0.05), indicating that the influence of comorbidities on prognosis was balanced between the two groups under the premise of standardized perioperative management.

The distribution of vascular occlusion sites between the two groups was not significantly different, including the MCA, ICA, BA segment (*P* > 0.05). Due to the current sample size, effective statistical comparisons cannot be temporarily made for fine subgroups such as the carotid artery T/I segment. Univariate logistic regression analysis also showed that vascular occlusion sites were not independent predictors of 90-day poor prognosis (*P* > 0.05), indicating that the influence of different vascular occlusion sites on the prognosis was balanced between the two groups, and did not affect the comparative results of BT and DMT.

Although the 7d NIHSS score of the BT group was slightly lower than that of the DMT group (Z = −0.86, *P* = 0.384), there was no statistically significant difference, suggesting that BT may have a potential advantage in short-term neurological function recovery, which may be related to the earlier reperfusion effect of intravenous thrombolysis in bridging therapy. The 90d NIHSS score of the two groups was not significantly different (*P* > 0.05), which was consistent with the results of 90d mRS, confirming that the short-term potential advantage of BT did not translate into a long-term significant difference. We chose mRS as the primary endpoint of long-term prognosis because it is more suitable for evaluating the overall functional independence of elderly stroke patients, while NIHSS is more focused on neurological deficit assessment (19, 23). In terms of long-term outcomes, no significant differences in the 90-day mRS scores were observed between the two groups, suggesting that both BT and DMT are comparably effective in restoring neurological function in the elderly. Despite ongoing debate in the literature regarding the relative benefits of BT and DMT on long-term functional recovery (24–26), bridging therapy may still offer early reperfusion advantages in patients with high thrombus burden or suboptimal response to intravenous thrombolysis (27). However, this benefit may not translate into improved outcomes in elderly patients, as seen in our study, warranting further investigation.

Multivariate analysis further identified puncture-to-treatment time, early NIHSS scores, and CRP levels at 7 days postoperatively as significant independent predictors of 90-day functional outcomes. These findings emphasize the importance of timely intervention and close monitoring of inflammatory markers post-thrombectomy. Specifically, shorter time from symptom onset to puncture was significantly associated with better outcomes, reinforcing the well-established principle that earlier reperfusion leads to reduced irreversible neurological damage (28) (29). According to the results of this study, the risk of adverse prognosis was 6.36 times that of patients with time > 3.25 h before puncture. Moreover, NIHSS scores at 24 h post-treatment emerged as an independent prognostic factor, reflecting that early neurological status strongly influences long-term recovery. Lower early NIHSS scores and rapid improvement typically indicate favorable outcomes. In this study, it was also found that patients with NIHSS score > 14.5 at 24 h after surgery had a risk of adverse prognosis 2.22 times higher than those with NIHSS score ≤ 14.5. Additionally, elevated CRP levels at day 7 were significantly associated with poorer functional prognosis. In addition, the study found that every 1 mg/L increase in CRP level on day 7 after surgery increased the risk of adverse prognosis by 2%, and the risk increased significantly when CRP level was > 28.175mg/L. As an acute-phase protein, CRP reflects the systemic inflammatory burden, and its persistent elevation may indicate ongoing tissue damage or secondary complications (30) (31). Given that all patients in this study were elderly, and aging is associated with heightened inflammatory sensitivity and reduced recovery potential (32), the impact of CRP levels on long-term outcomes is particularly pronounced in this population.

Despite the strong performance of the constructed prognostic model—which incorporates puncture time, NIHSS, and CRP, achieving an area under the ROC curve of 0.93—several limitations should be acknowledged. First, as a retrospective single-center study, selection bias and limited generalizability cannot be excluded. Second, despite balancing baseline characteristics using PSM, the inherent heterogeneity among elderly patients may still influence outcomes. Third, postoperative complications, rehabilitation course, and other concurrent treatments may confound the interpretation of early predictors. For instance, complications such as symptomatic intracranial hemorrhage or pneumonia within 7 days postoperatively may affect NIHSS dynamics, thereby attenuating its predictive power for long-term prognosis. Fourth, the sample size of this study was relatively small (22 patients in each group after PSM), which may reduce the statistical power of intergroup comparison and increase the probability of type II error. Although PSM was used to balance the baseline characteristics of the two groups and ensure the comparability, the small sample size still limits the generalization of the research results to a certain extent, and it is impossible to further subgroup analyze the influence of different vascular occlusion sites, comorbidity severity and other factors on the prognosis. As a supplementary and non-standard intervention in this study, CV8 acupoint patch may have certain effects on the prognosis and functional outcomes of the included patients. Although this intervention was applied consistently in the study population, such non-standard therapy might still affect the homogeneity of the overall treatment protocol and restrict the generalizability of the current results.

Additionally, several recent landmark trials, including DIRECT-MT and SWIFT-DIRECT, have compared the efficacy and safety of direct medical therapy (DMT) vs. bridging therapy (BT) for acute ischemic stroke. Although our study differs from these trials in baseline characteristics and study design, the overall findings are largely consistent, demonstrating that DMT yields comparable or superior clinical outcomes in selected patient populations. By integrating our results into a broader evidence base, this study further supports the growing body of evidence regarding the optimal revascularization strategy for acute ischemic stroke.

Therefore, the conclusions of this study should be interpreted with caution. Future research should conduct larger-scale, multicenter, prospective clinical trials with stricter intervention controls to further validate the findings and enhance the external validity of the results.

## Conclusion

In summary, both direct mechanical thrombectomy and bridging thrombectomy are effective in improving neurological outcomes in patients aged 75 years and older with acute ischemic stroke, with no significant differences in short- or long-term functional outcomes. However, BT may be associated with a heightened inflammatory response. Timeliness of treatment and postoperative inflammatory markers, particularly CRP, play crucial roles in determining long-term prognosis. This study provides valuable clinical evidence to guide treatment strategy selection in elderly AIS patients. Future research should further explore the differential impacts of revascularization strategies in this age group and investigate how integrating clinical and biomarker profiles can optimize individualized therapy.

## Data Availability

The original contributions presented in the study are included in the article/supplementary material, further inquiries can be directed to the corresponding author.
